# Complier-average causal effects for multivariate outcomes: an instrumental variable approach with application to health economics

**DOI:** 10.1186/1745-6215-16-S2-O44

**Published:** 2015-11-16

**Authors:** Karla DiazOrdaz, Angelo Franchini, Richard Grieve

**Affiliations:** 1 London School of Hygiene and Tropical Medicine, London, UK

## 

In randomised controlled trials that have non-compliance with the treatment assigned, policy makers require unbiased estimates of the causal effect of the treatment received. Instrumental variable (IV) approaches provide complier average causal effects (CACE) estimates. Common IV methods such as two-stage least squares (2SLS) have not been extended to settings with multivariate outcomes.

We propose a three-stage least squares (3SLS) regression approach, whereby estimates from the first stage regression of treatment received conditional on assignment, feed into a seemingly unrelated regression (SUR) system of equations that recognise the correlation between the outcomes. We also develop Bayesian IV approaches which jointly model the effects of random assignment on treatment received, and the bivariate outcome, which here is assume to be cost-effectiveness. We also apply 2SLS individually to each outcome, for comparison.

We consider the performance of these methods in a simulation study, where costs are assumed to follow Normal or Gamma distributions, to have positive and negative correlation with health outcomes, the instrument is strong (30% non-compliance) or weak (70% non-compliance), and the sample size, moderate (n=1,000) or small (n=100). We find that the proposed IV methods generally perform well. For example, in scenarios with Normally distributed cost data and a strong instrument, each method reports unbiased estimates. However, in these settings the 2SLS approach reports levels of Confidence Interval (CI) coverage that are above (positive correlation) and below (negative correlation) nominal levels. By contrast both the 3SLS and Bayesian methods report CI coverage close to nominal levels.

